# PD-1/PD-L1 Inhibitors versus Chemotherapy for Previously Treated Advanced Gastroesophageal Cancer: A Meta-Analysis of Randomized Controlled Trials

**DOI:** 10.1155/2021/3048974

**Published:** 2021-09-16

**Authors:** Zhong Maoxi, Xue Jinmin, Zeng Xiaozhu, Yue Yubing, Zhu Yuxi

**Affiliations:** ^1^Department of Oncology, The First Affiliated Hospital of Chongqing Medical University, Chongqing, China; ^2^Department of Oncology, Jinshan Hospital of The First Affiliated Hospital of Chongqing Medical University, Chongqing, China; ^3^Chongqing Clinical Cancer Research Center, Chongqing, China

## Abstract

Patients with advanced gastroesophageal cancer refractory to the previous regimen of chemotherapy suffered from poor prognosis without many effective treatment options. Immune checkpoint inhibitors (ICIs) provide promising efficacy, but the relevant clinical trials have offered controversial data. We performed this meta-analysis to compare the efficacy and safety of inhibitors against programmed cell death receptor 1 (PD-1) and its ligand PD-L1 versus chemotherapy as second or third-line therapy in patients with advanced gastroesophageal cancer. Six randomized controlled trials (RCTs) including 2,648 patients were included. The meta-analysis results indicated that both ORR (RR = 1.39, 95% CI: 0.85∼2.25, *P* = 0.188) and PFS (HR = 1.14, 95% CI: 0.88∼1.46, *P* = 0.316) were not significantly improved by ICIs compared with chemotherapy. However, the OS was significantly prolonged (HR = 0.85, 95% CI: 0.75–0.97, *P* = 0.018) in the ICIs group compared with chemotherapy. Subgroup analysis showed that ICIs provide statistically significant OS benefits over chemotherapy in PD-L1-positive, squamous cell carcinoma, Asia origin, esophageal cancer, second-line treatment, male, and aged 65 or older patients. Compared with chemotherapy, the TRAEs risk of ICIs was reduced by 33% (RR = 0.67, 95% CI: 0.62–0.73, *P* ≤ 0.001). And the risk of grades 3–5 of TRAEs was reduced by 60% (RR = 0.40, 95% CI: 0.33–0.49, *P* ≤ 0.001). Compared to chemotherapy, ICIs appeared to improve OS and were better tolerated in previously treated patients with advanced esophageal cancer. We recommend PD-1/PD-L1 inhibitors as an optimal treatment option for positive PD-L1 expression, squamous cell carcinoma, Asia origin, esophageal cancer, second-line treatment, male, and ≥65 years of age patients.

## 1. Introduction

Upper gastrointestinal cancer is one of the leading causes of cancer-related death in the world, with about 1.6 million new cases and 1.3 million deaths in 2018 [1]. Because of concealed incidence and rapid development, the prognosis of gastroesophageal cancer is really poor. Most of the patients tend to be diagnosed at advanced stages and lost the opportunity for operation. For these patients with advanced gastroesophageal cancer, combined chemotherapy based on 5-fluorouracil and platinum is the standard first-line treatment [2]. However, it is easy to develop drug resistance and result in disease progression after first-line treatment, while the efficacy of the following chemotherapy regime is not desirable with severe side effects. The patients who have failed in previous regimens are often in poor physical condition and are difficult to bear subsequent treatment. The medicines targeting epidermal growth factor receptor (EGFR), human epidermal growth factor receptor 2 (HER-2), and vascular endothelial growth factor (VEGF) showed effect on some patients, but the benefits are limited [3]. Therefore, how to improve the efficacy of patients with advanced gastroesophageal cancer refractory or intolerant to previous chemotherapy is an urgent problem. Recently, a number of clinical trials have shown that immune checkpoint inhibitors, represented by PD-1/PD-L1 inhibitors, exerted their potential in the posterior line treatment of advanced gastroesophageal cancer. However, there were still some trials without favorable outcomes. In addition, the correlation among pathological types, PD-L1 expression level, and curative effect, as well as the antitumor efficacy between ICIs and chemotherapy, is still worthy of further exploration. At present, there is still lack of meta-analysis of related randomized controlled trials. Thus, we performed this meta-analysis to integrate the efficacy, prognostic marker, and side effect of ICIs in published clinical trials of advanced esophageal cancer (EC), gastric cancer (GC), and gastroesophageal junction cancer (GEJC).

## 2. Materials and Methods

### 2.1. Search Strategy

According to the Population, Intervention, Comparison, Outcomes and Study (PICOS) design framework [4], we selected RCTs from multiple databases (PubMed, Cochrane Library, Web of Science, EMBASE, and CNKI database). The search terms included synonyms and medical subject headings (MeSH), and the Boolean operator (AND/OR) was used to combine the search words and search conditions. The two investigators searched these databases separately and screened out all the relevant literature up to October 2020. The specific search strategy is shown in Table 1.

### 2.2. Inclusion and Exclusion Criteria

Studies eligible for inclusion met all of the following criteria: (1) patients were clinically diagnosed as advanced EC/GC/GEJC and progressed after the failure of one or more chemotherapy regimens; (2) the trial group was treated with a single PD-1/PD-L1 inhibitor; (3) the control group was treated with chemotherapy; (4) the outcome index included clinical efficacy and survival analysis judged by RECIST criteria, and TRAEs were classified and graded; and (5) prospective RCTs.

The exclusion criteria were as follows: (1) repeated publication of data; (2) the outcome index was ambiguous or could not be merged; (3) non-RCTs.

### 2.3. Data Extraction and Quality Assessment

Two investigators independently reviewed the full text and evaluated the quality of the included literature. The literature authors, publication time, study design, number of patients, expression level of PD-L1, intervention measures, and outcome indicators were extracted and summarized. The HR of OS and PFS, ORR, TRAEs, and supplementary information was obtained from each eligible trial. The RevMan software provided by Cochrane Collaboration has a built-in Cochrane bias risk assessment tool and provides visual results (Copenhagen: The Nordic Cochrane Centre, The Cochrane Collaboration, 2014). According to Cochrane system Evaluation Manual [5], the included RCTs were evaluated in seven aspects: (1) random sequence generation; (2) allocation concealment; (3) blinding of participants and personnel; (4) blinding of outcome assessment; (5) incomplete outcome data; (6) selective reporting; and (7) other bias. Each item was divided into low risk, high risk, or risk unknown. Any discrepancies were resolved by discussion.

### 2.4. Statistical Analysis

Statistical software stata 14.2 was used for meta-analysis and publication bias test. For survival indicators (PFS and OS), HR was used to aggregate the statistics, and 95% confidence interval (CI) for estimating each point was calculated. RR and its corresponding 95% CI were used as effect indicators for ORR and TRAEs data. Statistical heterogeneity among the studies was assessed by the Cochran Q chi-square test and I^2^ statistic percentages [6]. When *I*^2^ ≤ 50% or *P* ≥ 0.05, it was considered that there was no significant heterogeneity, and the fixed effect model was used. Otherwise, when *I*^2^ > 50% or *P* < 0.1, it represented that there was heterogeneity among the studies; then, the random-effects model was used and possible sources of heterogeneity were sought. If a reasonable cause was found, a subgroup analysis was carried out. The difference was statistically significant when *P* < 0.05. Finally, we verified the credibility of the study through sensitivity analysis and published bias evaluation [5].

## 3. Results and Discussion

### 3.1. Search Results and Quality Evaluation

**Figure 1:**
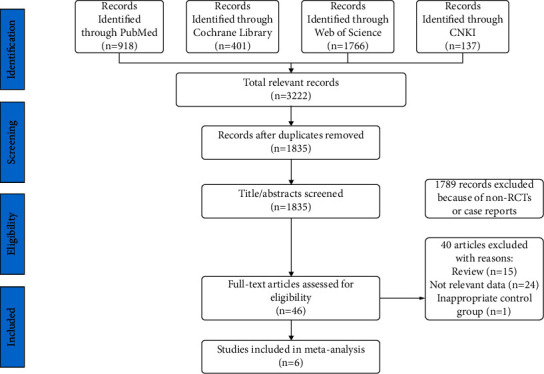
PRISMA flowchart showing the study selection process.

**Figure 2:**
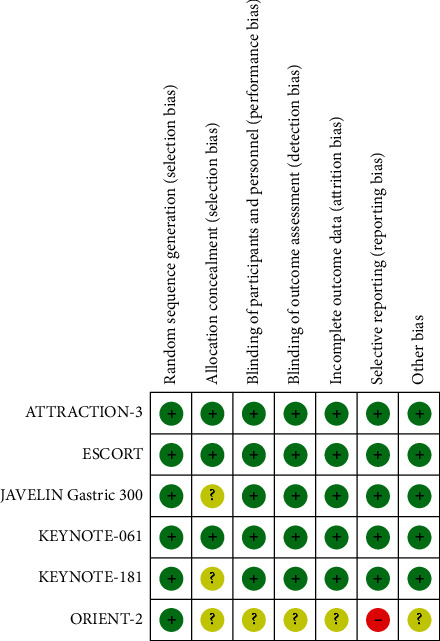
Literature quality evaluation (notes: green represents low risk bias, red represents high risk bias, and yellow represents unknown risk bias).

In this study, we identified a total of 3,222 related studies. The search terms and search strategy based on PICOS structure are shown in Table 1. After screening the titles and abstracts of these articles, we excluded duplicate or irrelevant literature. Then, there were 46 potentially eligible articles. After reading the full text, we eventually included 6 studies [7–12]. A flowchart of the above screening process is shown in Figure 1. Almost all the quality evaluations in the included literature were of low risk except for ORIENT-2 [12]. ORIENT-2 had a slightly higher risk of bias because this study was only presented by a meeting report, the specific study process was unknown, and the expression of PD-L1 in patients was not reported. The relevant quality evaluation is shown in Figure 2.

### 3.2. Characteristics of the Included Studies

The six studies included were prospective RCTs. Among them, ORIENT-2 [12] was a phase 2 clinical trial, and the rest were phase 3 clinical trials. In terms of research region, two of the six studies were from East Asia, and the other four studies were from many countries around the world. The characteristics of the included studies are shown in Table 2.

### 3.3. Objective Response Rate (ORR)

**Figure 3:**
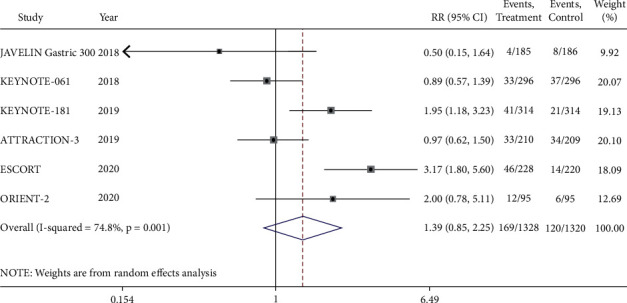
Meta-analysis results of objective response rate (ORR) between PD-1/PD-L1 inhibitor group and chemotherapy group.

We combined and analyzed six included studies comparing PD-1/PD-L1 inhibitors with chemotherapy for advanced gastroesophageal cancer. There was some heterogeneity among the studies (*I*^2^ = 74.8%, *P* < 0.1), so the random-effects model was used. The results showed that the short-term efficacy of anti-PD-1/PD-L1 in advanced gastroesophageal cancer was similar to that of chemotherapy, and there was no significant difference in ORR (RR = 1.39, 95% CI 0.85–2.25, *P* = 0.188) (Figure 3). In addition, we conducted a subgroup analysis according to tumor types and found that patients with the squamous subtype receiving PD-1/PD-L1 inhibitors showed significant ORR improvement compared with chemotherapy (RR = 1.89, 95% CI 1.03–3.44, *P* = 0.039), and the difference was statistically significant. However, as for the ORR of adenocarcinoma, there was no significant difference (RR = 0.88, 95% CI 0.60–1.29, *P* = 0.524) (Figure S1).

### 3.4. Progression-Free Survival (PFS)

**Figure 4:**
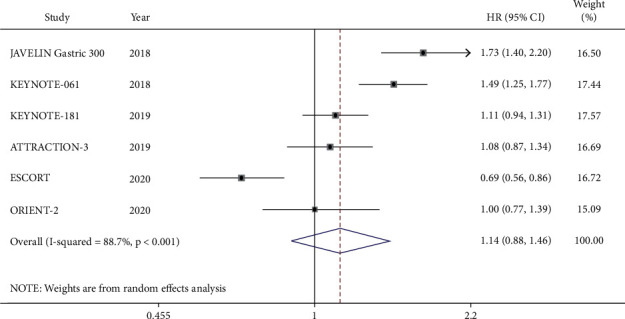
Meta-analysis results of progression-free survival (PFS) between the PD-1/PD-L1 inhibitor group and chemotherapy group.

The results of PFS showed that there was some heterogeneity among the six RCTs (*I*^2^ = 88.7%, *P* < 0.1), so the random-effects model was used for meta-analysis. The forest map showed that ICIs had no benefit in prolonging the PFS of advanced gastroesophageal cancer compared with that of chemotherapy (HR = 1.14, 95% CI 0.88–1.46, *P* = 0.316) (Figure 4). Subgroup analysis was performed across 6 RCTs. A survival benefit was not obtained in the squamous group for the PFS compared between two kinds of treatment (HR = 0.91, 95% CI 0.74–1.11, *P* = 0.329). On the other hand, for patients with adenocarcinoma, ICIs were less beneficial to PFS than that of chemotherapy (HR = 1.58, 95% CI 1.37–1.82, *P* ≤ 0.001) (Figure S2).

### 3.5. Overall Survival (OS)

**Figure 5:**
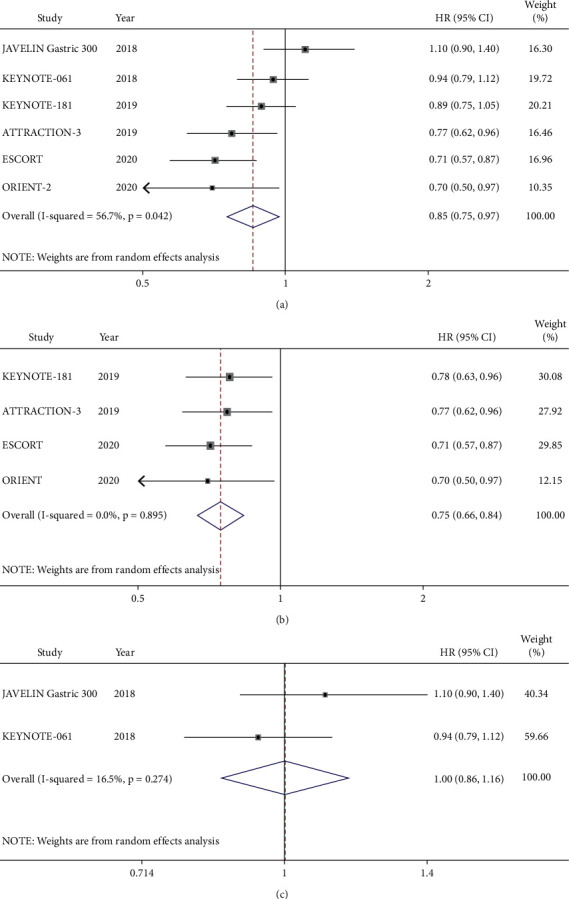
Meta-analysis results of overall survival (OS) between the PD-1/PD-L1 inhibitor group and chemotherapy group. (a) All patients. (b) Squamous cell carcinoma subgroup. (c) Adenocarcinoma subgroup.

As shown in the forest plot, the OS among the six RCTs including 2,648 patients was compared between ICIs and chemotherapy (Figure 5(a)). Due to some heterogeneity between studies (*I*^2^ = 56.7%, *P* < 0.1), the random-effects model was used. ICIs did prolong the OS of the patients compared to that of chemotherapy (HR = 0.85, 95% CI 0.75–0.97, *P* = 0.018). Subgroup analysis showed that the ICIs significantly increased the OS of squamous cell carcinoma (HR = 0.75, 95% CI 0.66–0.84, *P* ≤ 0.001) (Figure 5(b)). However, there was no difference of the OS for adenocarcinoma treated by two treatment regimens (HR = 1.00, 95% CI 0.86–1.16, *P* = 0.984) (Figure 5(c)). In addition, the analysis of the pooled data indicated that ICIs significantly improved the OS of the EC compared with that of chemotherapy (HR = 0.79, 95% CI 0.70–0.88). As for GEJC, ICIs also showed some clinical benefit, although there was no statistically difference (HR = 0.71, 95% CI 0.51–1.00). On the other hand, these RCTs did not provide enough data to support the same conclusion for GC. According to the PD-L1 expression status, ICIs remarkably reduced the risk of death of PD-L1-positive patients compared that of chemotherapy (HR = 0.73, 95% CI 0.63–0.84, *P* ≤ 0.001), while there was no survival advantage for PD-L1-negative patients (HR = 1.00, 95% CI 0.81–1.24, *P* = 0.998). Furthermore, the pooled results showed that PD-1/PD-L1 inhibitors had significant benefits in the subgroups of second-line application, Asian region, male, and aged 65 or older (Table 3).

### 3.6. Treatment-Related Adverse Events (TRAEs)

**Figure 6:**
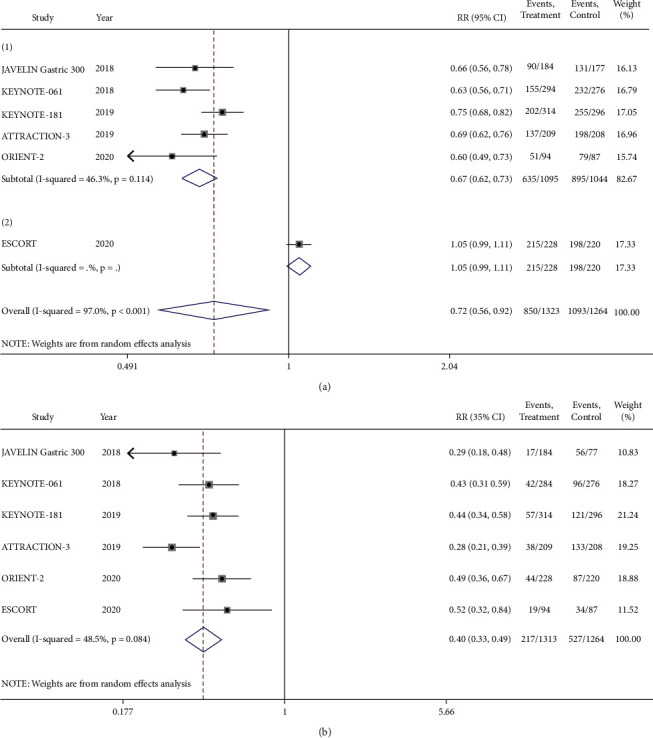
TRAEs of the PD-1/PD-L1 inhibitor group versus the chemotherapy group. (a) Any grade of TRAEs. (b) Grades 3–5 of TRAEs.

We compared all the TRAEs between the research group and the control group. After exclusion of the ESCORT study which brought heterogeneity, we found that the incidence of TRAEs on immunotherapy was significantly lower than that on chemotherapy (RR = 0.67, 95% CI 0.62–0.73, *P* ≤ 0.001) (Figure 6(a)). For severe (grades 3–5) TRAEs, the risk of immunotherapy was 60% lower than that of chemotherapy. The sensitivity analyses of the study (RR = 0.40, 95% CI 0.33–0.49, *P* ≤ 0.001) (Figure 6(b)). The most common TRAEs in both groups were fatigue, nausea, diarrhea, anemia, neutrophil count decreased, white blood cell (WBC) count decreased, and bone marrow toxicity, which were significantly lower in the immunotherapy group both in any grade and in grades 3–5 of TRAEs (Tables S1 and S2). The incidence of hypothyroidism increased significantly for ICIs, but without grades 3–5 of hypothyroidism. The risk of pulmonary infection in the immunotherapy group was also higher than that in the chemotherapy group, but the difference was not statistically significant. The incidence of death caused by TRAEs was similar between the two groups.

### 3.7. Publication Bias Assessment and Sensitivity Analyses

Due to the limited number of included clinical studies (*n* < 10), we did not detect publication bias. Sensitivity analysis was performed by excluding the literature one by one. And the sensitivity analysis showed that there was no significant difference between the primary results after the exclusion of the study and the previous results, indicating that the sensitivity was low, and the results were robust and credible (Table 4). Among them, the results of individual studies had some influence on the effect sizes of PFS and OS in the meta-analysis. However, in general, we reached more comprehensive conclusions on the efficacy of immunotherapy in this large group of patients with gastroesophageal cancer by expanding the sample size for combined analysis.

## 4. Discussion

In this paper, we compared the efficacy and safety of ICIs against chemotherapy beyond the first line of advanced gastroesophageal cancer. First, for the short-term efficacy, ORR and PFS of the immunotherapy group were not significantly improved compared with that of the chemotherapy group. Regarding ORR, there was a tendency that ICIs were superior to chemotherapy in the squamous cell carcinoma subgroup, however, there was no statistical difference. And, there was no difference in the PFS. On the other hand, for the adenocarcinoma, there was no difference between ICIs and chemotherapy in ORR, and the PFS of ICIs was even inferior to chemotherapy. However, the ICIs were superior to chemotherapy in the long-term efficacy, especially for squamous cell carcinoma. In addition, our data suggested that patients with positive PD-L1 expression, of Asian regions, with esophageal tumors, and scheduled for second-line treatment; male patients; and patients ≥65 years of age could benefit from ICIs, instead of chemotherapy.

Gastroesophageal cancer is a disease with extensive heterogeneity among different histologic types and tumor sites. Studies have shown that esophageal squamous cell carcinoma has unique molecular characteristics, while esophageal adenocarcinoma and gastric adenocarcinoma have more similarities [13]. Our subgroup analysis showed that the histological type was the main source of heterogeneity among studies. There were significant differences in the effect size of the primary outcomes between patients with squamous cell carcinoma and patients with adenocarcinoma, and the subgroup analysis showed that the histology was the main factor affecting PFS and OS. Therefore, it might be better to investigate adenocarcinoma and squamous cell carcinoma separately in clinical trials of gastroesophageal cancer. The results of subgroup analysis confirmed that PD-L1 was a good predictor of ICIs for gastroesophageal cancer. PD-L1 overexpression is more common in esophageal squamous cell carcinoma (ESCC) (41%), compared with gastric adenocarcinoma (GAC) (10%), esophageal adenocarcinoma (EAC) (9%), and gastroesophageal junction adenocarcinoma (GEJAC) (8%), while high microsatellite instability (MSI-H) and high tumor mutational burden (TMB-H) are rare in gastroesophageal cancer [13]. This may be one of the reasons for the better benefits of immunotherapy in squamous cell carcinoma. Due to the different detection methods of PD-L1 expression in different studies, such as Combined Positivity Score (CPS) and Tumor Proportion Score (TPS), we were unable to further analyze the relationship between PD-L1 expression level and efficacy.

As for the third-line and the subsequent treatment, due to insufficient data, we were unable to conduct a combined analysis. Only the JAVELIN Gastric 300 study was involved in this paper. Four single-arm studies on the third-line and subsequent treatment of EC, including JapicCTI-No.142422 [14], Keynote-028 [15], Keynote-180 [16], and NCT02742935 [17], showed that patients treated with PD-1 inhibitors achieved an ORR of 14.3%–33.3%, among which two studies reported the mOS of 7.0 months and 10.8 months, respectively. The related studies of GC and GEJC, including JAVELIN Gastric 300 [7], ATTRACTION-2 [18], and KEYNOTE-059 cohort 1 [19], showed that the patient's ORR was 3.2%–16.4% and mOS was 4.2–5.6 months. Therefore, in general, PD-1/PD-L1 inhibitors were effective in the third-line and later application of EC, but not satisfactory in GC and GEJC. Overall, immunotherapy was well tolerated for patients with third-line and subsequent palliative treatment. The incidence of grades 3–5 of TRAEs reported in EC was 10%–17% [11, 20], and that of GC and GEJC was 9.2%–23.3% [11, 20].

In this meta-analysis, we observed that the ORR of immunotherapy was consistent with that of OS for both squamous cell and adenocarcinoma. However, in the immunotherapy group, the tendency of PFS and OS was not the same. We suggest that the reason for this contradiction may be related to the antitumor response of ICIs as opposed to chemotherapy. First of all, one of the major differences between immunotherapy and cytotoxic therapy is the lag of response [21]. For example, ATTRACTION-3 [10] showed that the median time of onset of ICIs was later than chemotherapy (2.6 months vs. 1.5 months). Secondly, immunotherapy shows a long duration of response (DOR). KEYNOTE-061 [8], ATTRACTION-3 [10], ESCORT [11], and ORIENT-2 [12] showed that the median DOR of immunotherapy was longer than that of chemotherapy (18.0 months vs. 5.5 months, 6.9 months vs. 3.9 months, 7.4 months vs. 3.4 months, 8.3 months vs. 6.2 months, respectively). There was an intersection point of the Kaplan–Meier curve of PFS in all the second-line trails in this paper, except for the JAVELIN Gastric 300 study for the third-line application. The PFS curve of the immunotherapy group started below the chemotherapy group, crossed at about 4 months to 10 months, and then appeared above the chemotherapy group. This means that patients who were effective in immunotherapy got a long durable response and progress more slowly than those treated by chemotherapy. The five studies [8–12] of second-line trails showed that the 6-month OS rate of the immune group was similar to that of the chemotherapy group, while the 1-year OS rate and 18-month OS rate were significantly higher than those of the chemotherapy group, which were 1.3 to 1.8 times and 1.4 to 1.8 times, respectively. Therefore, we propose that the rates of OS at 6 months, 1 year, and 18 months are a reliable indicator for immunotherapy.

It is worth mentioning that although ICIs have shown promising efficacy in advanced gastroesophageal cancer, a specific pattern of tumor response, known as the hyperprogressive disease (HPD), has been observed clinically, as well as in other solid tumors. HPD is reported to occur in about 10% to 21% of advanced gastric cancer patients treated with PD-1 inhibitors [22, 23]. At present, the mechanism of this atypical reaction is still under exploration. Studies have shown that the development of HPD in advanced GC patients treated with PD-1 inhibitors is related to the proliferation of tumor-infiltrating FoxP3highCD45RA−CD4 + T cells [regulatory T cells (Tregs)] [22]. This suggests that Tregs might be involved in the treatment of PD-1/PD-L1 blockade and PD-1/PD-L1 axis, thus regulating the antitumor immune response. Experiments in humans and mice have found that PD-1 blockade can enhance the activation of both Tregs and conventional T (Tconv) cells by increasing the signal intensity of T cell receptor (TCR) and CD28. And the proliferation of Tregs may contribute to tumor progression even in the presence of an effective antitumor immune response mediated by Tconv cells, and the final outcome depends on which response dominates [22]. In hepatocellular carcinoma (HCC), significant accumulation of Tregs and consumption of proinflammatory T cells were also observed to be closely related to tumor development and spread [24]. Therefore, monitoring Tregs in tumor and peripheral blood may be an important predictor of the efficacy of ICI. At the same time, inhibiting the proliferation of tumor-infiltrating Tregs may reduce the incidence of HPD.

In terms of safety, immunotherapy had better tolerance, with a 33% and 60% reduction in the risk of grades 1–5 of TRAEs and grades 3–5 of TRAEs compared with chemotherapy, respectively. The inevitable autoimmune attack also occurs when the immune system is activated to fight cancer [25, 26]. Nonetheless, most immune-related adverse events are controllable by the withdrawal of ICIs and steroids [21]. A new and special adverse reaction, reactive cutaneous capillary endothelial proliferation (RCCEP), was reported only in the ESCORT study, with an incidence of 79%, mainly grades 1-2. RCCEP was also observed in other studies of camrelizumab [20]. Interestingly, the analysis showed that RCCEP was associated with higher ORR and longer OS [11, 20]. Moreover, in the ORIENT-2 [12] study, a lower neutrophil to lymphocyte ratio (NLR <3) at baseline and week 6 was found to be associated with a longer OS. According to the previous report [27], different types of ICIs have slightly different molecular mechanisms, which may relate differences in TRAEs. Attention should be paid to the observation and analysis of the relationship between TRAEs and tumor response.

There are still some shortcomings of this study as follows: (1) there is insufficient data to support the benefits of ICIs compared with chemotherapy in third-line and subsequent applications; (2) due to different detection methods for PD-L1 expression, it is difficult to conduct an in-depth analysis of the relationship between the expression level of PD-L1 and efficacy; and (3) the number of studies included is limited, so we could not perform a subgroup analysis of PD-1 inhibitors and PD-L1 inhibitors, respectively.

## 5. Conclusions

In summary, PD-1/PD-L1 inhibitors appear to improve OS and have better tolerance compared to chemotherapy in the recurrence of advanced gastroesophageal carcinoma, especially for positive PD-L1 expression, squamous cell carcinoma, Asian region, esophageal cancer, second-line application, male, and 65 years of age or older patients. Due to the different antitumor response patterns of PD-1/PD-L1 inhibitors and cytotoxic drugs, more reasonable indicators, such as survival at some time point, are necessary for efficacy evaluation. More clinical trials are needed to concern about the different histology, PD-L1 expression level, and multiple lines treatment.

## Figures and Tables

**Table 1:** Search terms and search strategy based on PICOS structure.

Population	Intervention	Comparison	Outcomes	Study design	Combining search terms
Patients with advanced gastroesophageal cancer who progressed after the failure of one or more chemotherapy regimens	PD-1/PD-L1 inhibitor monotherapy	Chemotherapy	ORR, PFS, OS, TRAEs	Randomized controlled trial	Column 1 AND Column 2 AND Column 3
“Esophageal OR gastric OR gastroesophageal junction” AND “cancer OR carcinoma OR neoplasm OR tumor OR adenocarcinoma”	PD-1 OR PD-L1 OR immune checkpoint inhibitor OR immunotherapy OR pembrolizumab OR nivolumab OR avelumab OR atezolizumab OR durvalumab OR camrelizumab OR SHR-1210 OR toripalimab OR sintilimab OR tislelizumab			Study OR trial OR clinical trial OR randomized clinical trial OR randomized controlled trial OR randomized controlled clinical trial	

**Table 2:** Characteristics of eligible studies included in the meta-analysis.

Trial	Geographic area	Tumor type	Year	Design	Medication	No. of patients	Clinical stage	Gender (M/F)	Median age (y)	PD-L1-positive patients	Line
JAVELIN Gastric 300	Europe, Asia, North America, and the rest of the world	GEJC/GC	2018	Phase 3	Avelumab (10 mg/kg q2w) vs. chemotherapy (paclitaxel/irinotecan)	AVE: 185 CHE: 186	NA	AVE: 140/45 CHE: 127/59	AVE: 59 CHE: 61	AVE: 46/157 (29.3%) CHE: 39/160 (24.4%)	2–3 (line3, 86%)
KEYNOTE-061	Europe, Asia, North America, and the rest of the world	GEJC/GC	2018	Phase 3	Pembrolizumab (200 mg q3w) vs. chemotherapy (paclitaxel)	PEM: 296 CHE: 296	NA	PEM: 202/94 CHE: 208/88	PEM: 62.5 CHE: 60.0	PEM: 196/295 (66%) CHE: 199/295 (67%)	2
KEYNOTE-181	Asia and the rest of the world	EC/GEJC	2019	Phase 3	Pembrolizumab (200 mg q3w) vs. chemotherapy (paclitaxel/docetaxel/irinotecan)	PEM: 314 CHE: 314	NA	PEM: 273/41 CHE: 271/43	PEM: 63 CHE: 62	PEM: CPS≥10 (84); CPS<10 (89) CHE: CPS≥10 (84); CPS<10 (93)	2
ATTRACTION-3	Europe, East Asia, and the USA	ESCC	2019	Phase 3	Nivolumab (240 mg q2w) vs. chemotherapy (paclitaxel/docetaxel)	NIV: 210 CHE: 209	NIV : II-III: 8; IV: 94; CHE : II–III: 13; IV: 100	NIV: 179/31 CHE: 185/24	NIV: 64 CHE: 67	NIV: 101/210 (48%) CHE: 102/209 (49%)	2
ESCORT	East Asia	ESCC	2020	Phase 3	Camrelizumab (200 mg q2w) vs. chemotherapy (docetaxel/irinotecan)	CAM: 228 CHE: 220	NA	CAM: 208/20 CHE: 192/28	CAM: 60 CHE: 60	CAM: 93/222 (42%) CHE: 98/216 (45%)	2
ORIENT-2	East Asia	ESCC	2020	Phase 2	Sintilimab (200 mg q3w) vs. chemotherapy (paclitaxel/irinotecan	SIN: 95 CHE: 95	SIN : III:7; IV:86 CHE : III: 6; IV: 89	SIN: 88/7 CHE: 84/11	SIN: 58.8 CHE: 59.4	NA	2

**Table 3:** Subgroup analysis of OS (PD-1/PD-L1 inhibitors vs. chemotherapy).

Subgroup	Total no. of studies	Total no. of patients	HR	95% CI
*Histological type*				
SCC	4	1458	0.75	0.66∼0.84
ACC	2	872	1.00	0.86∼1.16

*Tumor site*				
EC	4	1685	0.79	0.70∼0.88
GEJC	2	246	0.71	0.51∼1.00
GC	2	520	1.07	0.82∼1.40

*Line*				
2	5	2186	0.82	0.73∼0.92
3∗	1	371	1.10	0.90∼1.40

*PD-L1 expression status*				
Positive	5	1096	0.73	0.63∼0.84
Negative	4	890	1.000	0.81∼1.24

*ECOG performance status*				
0	4	606	0.94	0.69∼1.28
1	4	1026	0.79	0.62∼1.02

*Sex*				
Male	4	1217	0.83	0.73∼0.93
Female	4	316	0.84	0.51∼1.38

*Age at baseline, years*				
<65	4	989	0.82	0.64∼1.05
≥65	4	644	0.83	0.70∼0.99

*Region*				
Asia	6	1592	0.76	0.65∼0.88
Non-Asia	3	524	0.90	0.71∼1.16

Notes: ∗ The data of line 3 came from all the participants in the JAVELIN Gastric 300 study. This RCT focused on second-line and third-line applications, of which 86% were third-line applications.

**Table 4:** The sensitivity analyses of the study.

Sensitivity analyses	No. of studies	RR (95%CI)			HR (95%CI)		
		No. of patients	ORR	No. of patients	PFS	No. of patients	OS
Total studies	6	2648	1.39 (0.85∼2.25)	2648	1.14 (0.88∼1.46)	2648	0.85 (0.75∼0.97)
JAVELIN Gastric 300 excluded	5	2277	1.55 (0.94∼2.55)	2277	1.05 (0.81∼1.35)	2277	0.82 (0.73∼0.92)
KEYNOTE-061 excluded	5	2056	1.54 (0.88∼2.71)	2056	1.07 (0.81∼1.43)	2056	0.83 (0.71∼0.98)
KEYNOTE-181 excluded	5	2020	1.28 (0.71∼2.28)	2020	1.4 (0.83∼1.58)	2020	0.84 (0.71∼1.00)
ATTRACTION-3 excluded	5	2229	1.51 (0.83∼2.72)	2229	1.15 (0.85∼1.56)	2229	0.87 (0.75∼1.10)
ESCORT excluded	5	2200	1.16 (0.77∼1.76)	2200	1.26 (1.04∼1.53)	2200	0.89 (0.78∼1.01)
ORIENT-2 excluded	5	2458	1.31 (0.76∼2.25)	2458	1.16 (0.87∼1.55)	2458	0.87 (0.76∼1.00)

## Data Availability

All the data generated or analyzed during this study are included in this published article.

## Supplementary Materials

Supplementary Materials Supplementary materials include Figures S1-S2 and Tables S1-S2 (see supplementary documents for details). Figure S1: Forest plot of risk ratios for objective response rate (ORR) between PD-1/PD-L1 inhibitors and chemotherapy/placebo in subgroups: (a) ORR in the squamous cell carcinoma subgroup; (b) ORR in the adenocarcinoma subgroup. Figure S2: Forest plot of hazard ratios for progression-free survival (PFS) between PD-1/PD-L1 inhibitors and chemotherapy/placebo in subgroups: (a) PFS in the squamous cell carcinoma subgroup; (b) PFS in the adenocarcinoma subgroup. Table S1: Any grade treatment-related adverse events of the PD-1/PD-L1 inhibitor group versus the chemotherapy group. Table S2: Grades 3–5 of treatment-related adverse events of the PD-1/PD-L1 inhibitor group versus the chemotherapy group.
