# Supplementary material for: PD-1/PD-L1 Inhibitors versus Chemotherapy for Previously Treated Advanced Gastroesophageal Cancer: A Meta-Analysis of Randomized Controlled Trials
Source: J Oncol. 2021 Sep 16;2021:3048974. doi: 10.1155/2021/3048974 (PMC8463210; doi:10.1155/2021/3048974)
Supplement: Supplementary Materials — include Figures S1-S2 and Tables S1-S2 (see supplementary documents for details). Figure S1: Forest plot of risk ratios for objective response rate (ORR) between PD-1/PD-L1 inhibitors and chemotherapy/placebo in subgroups: (a) ORR in the squamous cell carcinoma subgroup; (b) ORR in the adenocarcinoma subgroup. Figure S2: Forest plot of hazard ratios for progression-free survival (PFS) between PD-1/PD-L1 inhibitors and chemotherapy/placebo in subgroups: (a) PFS in the squamous cell carcinoma subgroup; (b) PFS in the adenocarcinoma subgroup. Table S1: Any grade treatment-related adverse events of the PD-1/PD-L1 inhibitor group versus the chemotherapy group. Table S2: Grades 3–5 of treatment-related adverse events of the PD-1/PD-L1 inhibitor group versus the chemotherapy group. [file 3048974.f1.zip › 20210908Supplementary Information (supplementary Fig and Table).docx]

**
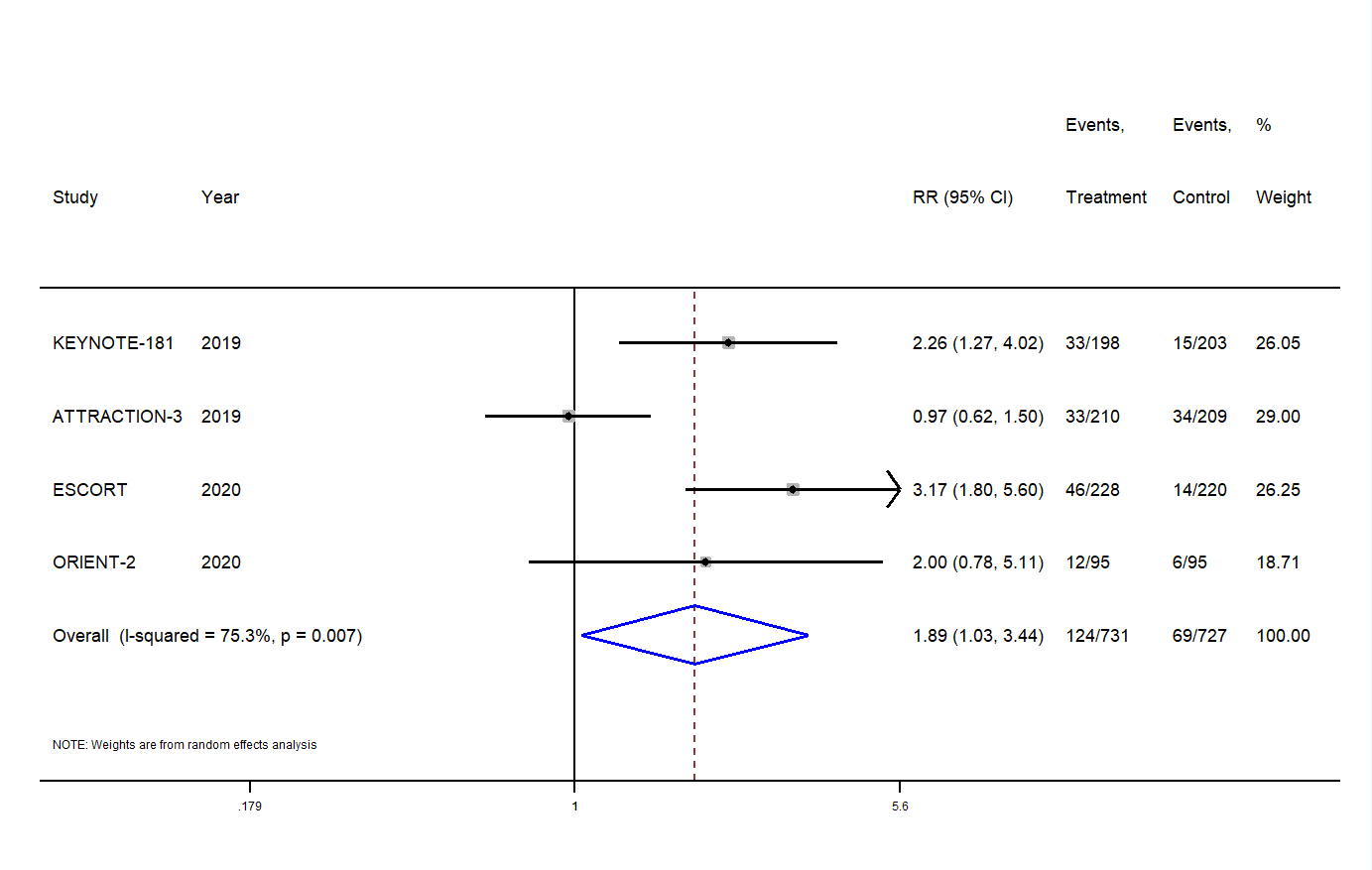
**

Figure S1a


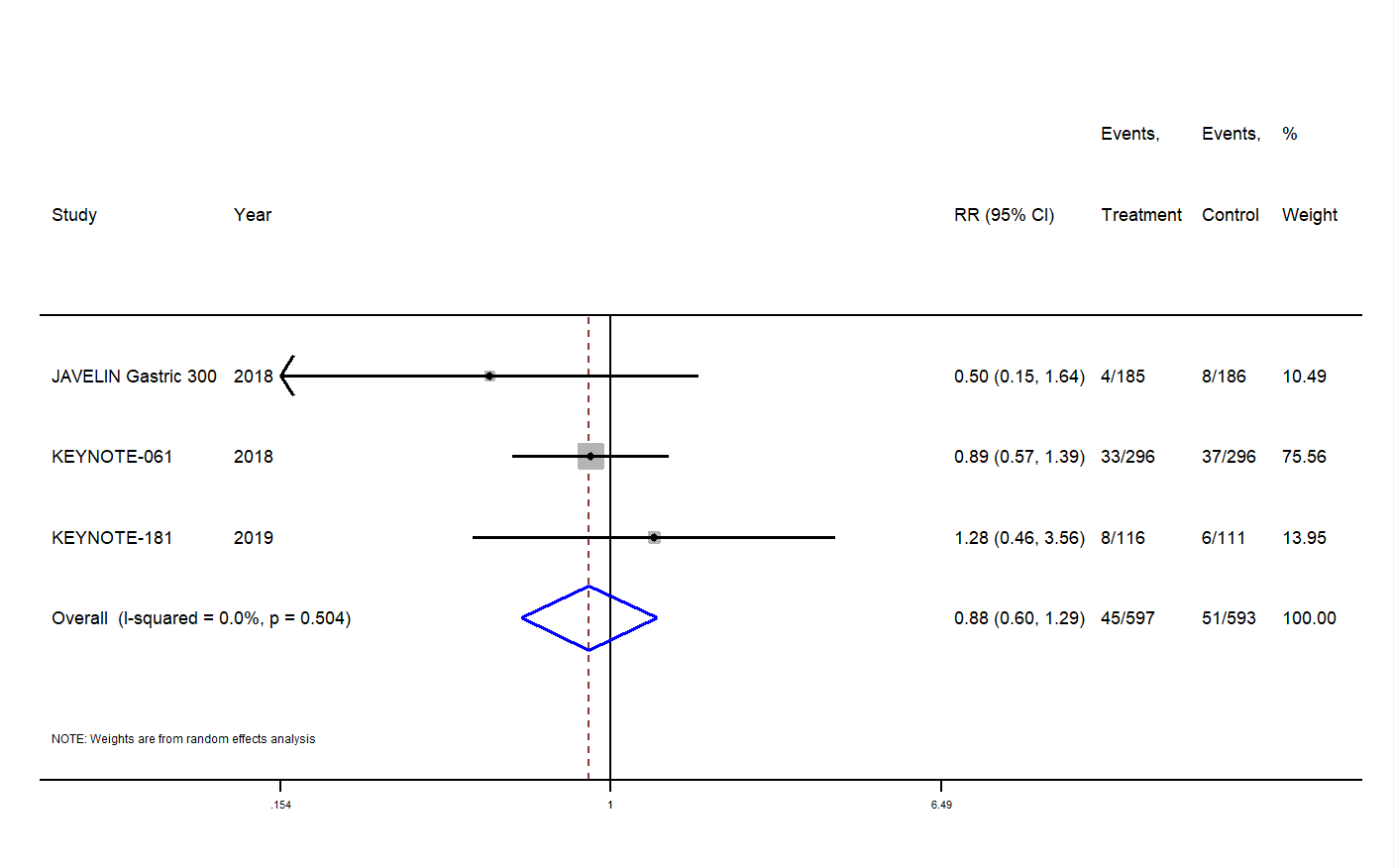


Figure S1b

Figure S1: Forest plot of risk ratios for objective response rate (ORR) between PD-1/PD-L1 inhibitors and chemotherapy in subgroups. (a) ORR in the squamous cell carcinoma subgroup. (b) ORR in the adenocarcinoma subgroup.


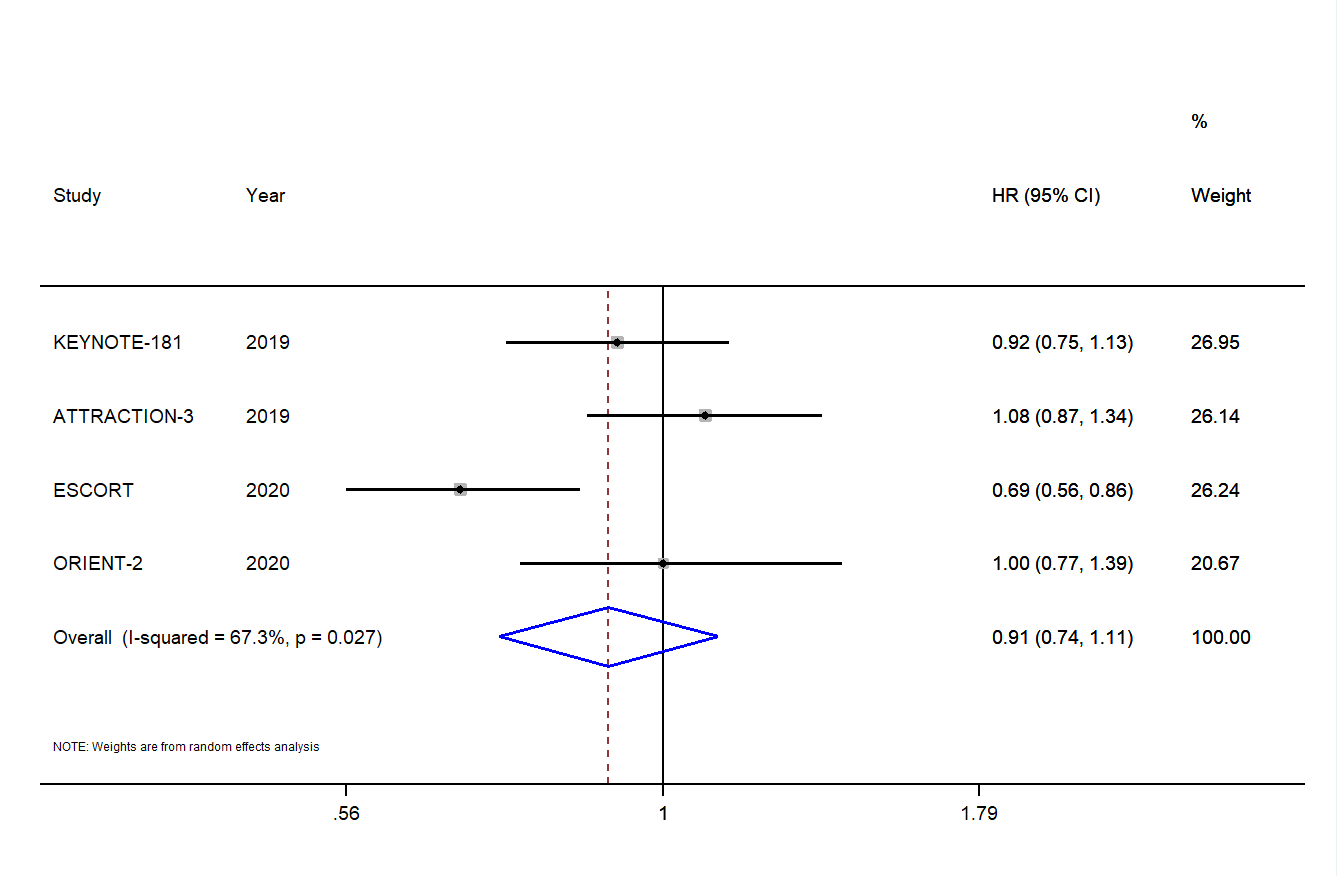


Figure S2a


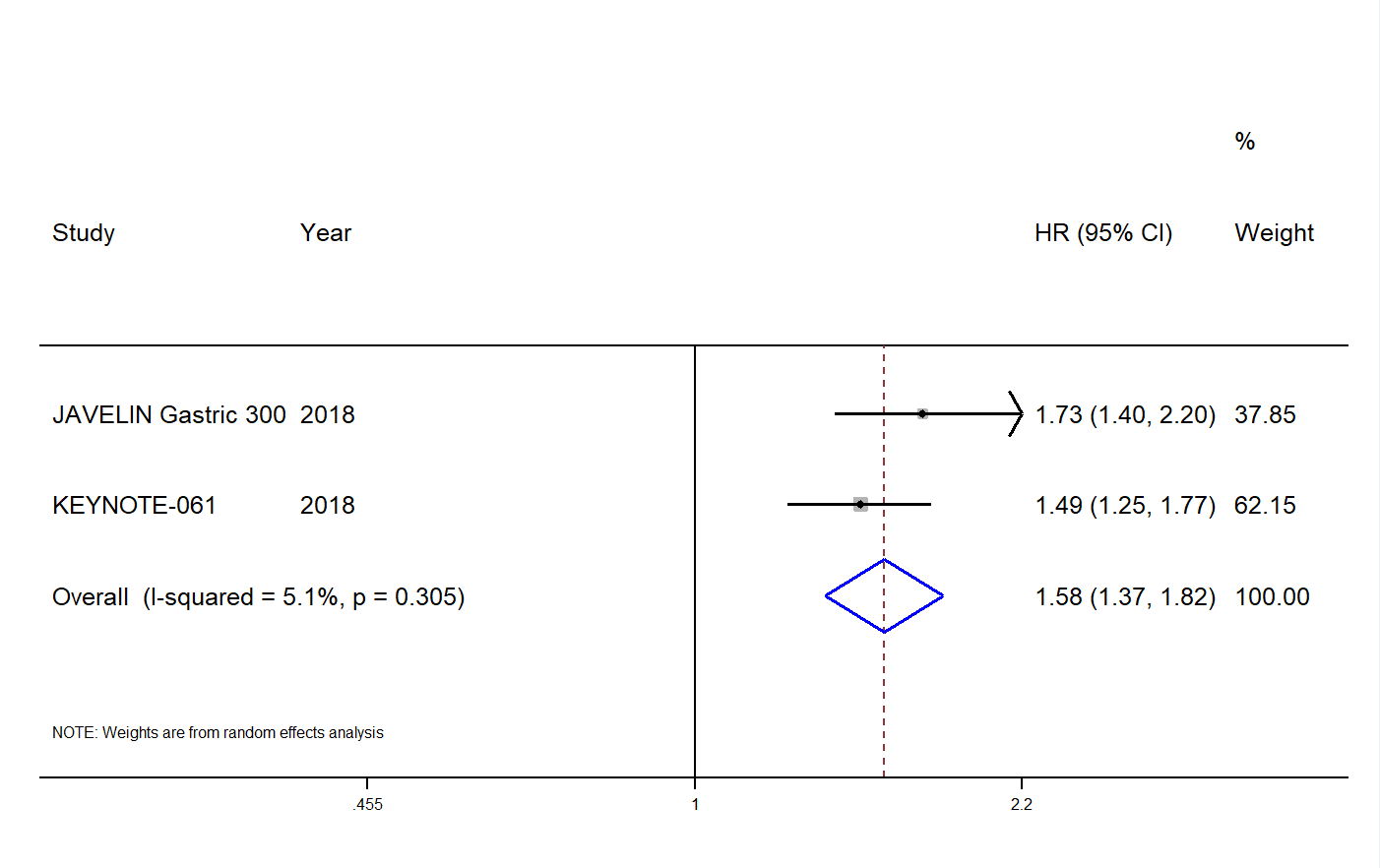


Figure S2b

Figure S2: Forest plot of hazard ratios for progression-free survival (PFS) between PD-1/PD-L1 inhibitors and chemotherapy in subgroups. (a) PFS in the squamous cell carcinoma subgroup. (b) PFS in the adenocarcinoma subgroup.

Table S1: Any grade treatment-related adverse events of the PD-1/PD-L1 inhibitor group vs. the chemotherapy group.

| TRAEs | No. of studies | No. of patients | RR | Min  95%CI | Max 95%CI | P-value |
| --- | --- | --- | --- | --- | --- | --- |
| Fatigue | 5 | 2139 | 0.48 | 0.38 | 0.61 | ≤0.001 |
| Nausea | 5 | 2406 | 0.19 | 0.10 | 0.34 | ≤0.001 |
| Diarrhea | 6 | 2587 | 0.30 | 0.17 | 0.54 | ≤0.001 |
| Anemia | 6 | 2587 | 0.16 | 0.09 | 0.26 | ≤0.001 |
| Neutrophil count decreased | 6 | 2587 | 0.07 | 0.03 | 0.14 | ≤0.001 |
| WBC count decreased | 5 | 2017 | 0.08 | 0.04 | 0.18 | ≤0.001 |
| Bone marrow toxicity | 2 | 629 | 0.05 | 0.01 | 0.40 | ≤0.001 |
| Hypothyroidism | 4 | 1809 | 7.85 | 4.14 | 14.86 | ≤0.001 |
| Lung infection | 3 | 1199 | 3.08 | 0.22 | 43.16 | 0.404 |
| Events leading to death | 4 | 1836 | 0.89 | 0.38 | 2.08 | 0.786 |

Table S2: Grade 3-5 treatment-related adverse events of the PD-1/PD-L1 inhibitor group vs. the chemotherapy group.

| TRAEs | No. of studies | No. of patients | RR | Min 95%CI | Max  95%CI | P-value |
| --- | --- | --- | --- | --- | --- | --- |
| Fatigue | 5 | 2139 | 0.46 | 0.20 | 1.07 | 0.072 |
| Nausea | 5 | 2406 | 0.17 | 0.05 | 0.59 | 0.005 |
| Diarrhea | 6 | 2587 | 0.31 | 0.15 | 0.66 | 0.002 |
| Anemia | 6 | 2587 | 0.28 | 0.15 | 0.53 | ≤0.001 |
| Neutrophil count decreased | 5 | 1977 | 0.04 | 0.01 | 0.10 | ≤0.001 |
| WBC count decreased | 4 | 1407 | 0.03 | 0.01 | 0.11 | ≤0.001 |
| Bone marrow toxicity | 2 | 629 | 0.08 | 0.01 | 0.60 | 0.014 |
| Hypothyroidism | 4 | 1809 | - | - | - | - |
| Lung infection | 3 | 1199 | 1.91 | 0.17 | 21.96 | 0.605 |
