# Supplementary material for: PD-1/PD-L1 Inhibitors versus Chemotherapy for Previously Treated Advanced Gastroesophageal Cancer: A Meta-Analysis of Randomized Controlled Trials
Source: J Oncol. 2021 Sep 16;2021:3048974. doi: 10.1155/2021/3048974 (PMC8463210; doi:10.1155/2021/3048974)
Supplement: Supplementary Materials — include Figures S1-S2 and Tables S1-S2 (see supplementary documents for details). Figure S1: Forest plot of risk ratios for objective response rate (ORR) between PD-1/PD-L1 inhibitors and chemotherapy/placebo in subgroups: (a) ORR in the squamous cell carcinoma subgroup; (b) ORR in the adenocarcinoma subgroup. Figure S2: Forest plot of hazard ratios for progression-free survival (PFS) between PD-1/PD-L1 inhibitors and chemotherapy/placebo in subgroups: (a) PFS in the squamous cell carcinoma subgroup; (b) PFS in the adenocarcinoma subgroup. Table S1: Any grade treatment-related adverse events of the PD-1/PD-L1 inhibitor group versus the chemotherapy group. Table S2: Grades 3–5 of treatment-related adverse events of the PD-1/PD-L1 inhibitor group versus the chemotherapy group. [file 3048974.f1.zip › Figure S2a.pdf]

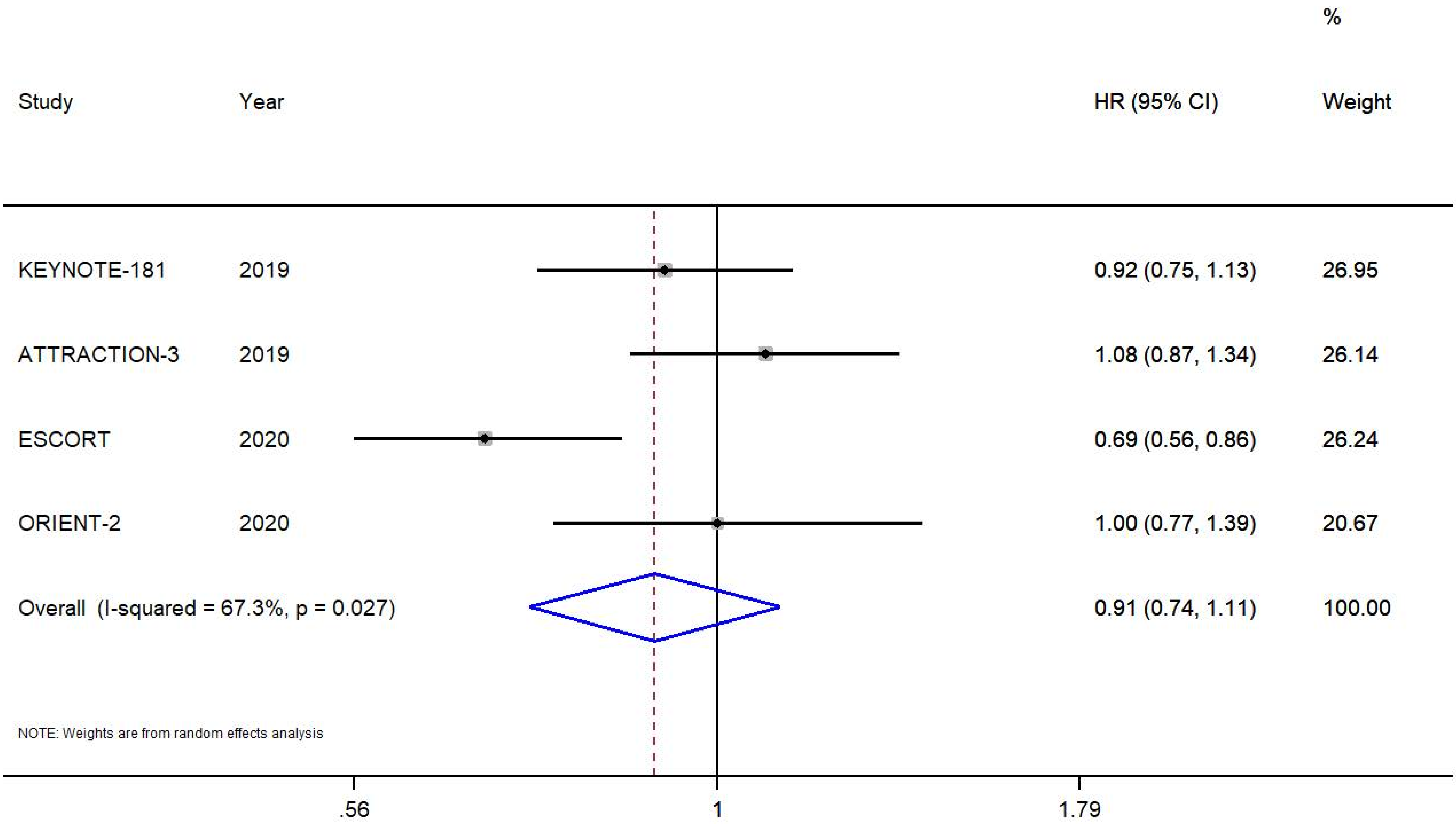
%
Study
Year
HR (95% CI)
Weight
KEYNOTE-181
2019
0.92 (0.75, 1.13)
26.95
ATTRACTION-3
2019
1.08 (0.87, 1.34)
26.14
ESCORT
2020
0.69 (0.56, 0.86)
26.24
ORIENT-2
2020
1.00 (0.77, 1.39)
20.67
Overall (I-squared = 67.3%, p = 0.027)
0.91 (0.74, 1.11)
100.00
NOTE: Weights are from random effects analysis
.56
1
1.79
