# Supplementary figures and images for: PD-1/PD-L1 Inhibitors versus Chemotherapy for Previously Treated Advanced Gastroesophageal Cancer: A Meta-Analysis of Randomized Controlled Trials
Source: J Oncol. 2021 Sep 16;2021:3048974. doi: 10.1155/2021/3048974 (PMC8463210; doi:10.1155/2021/3048974)

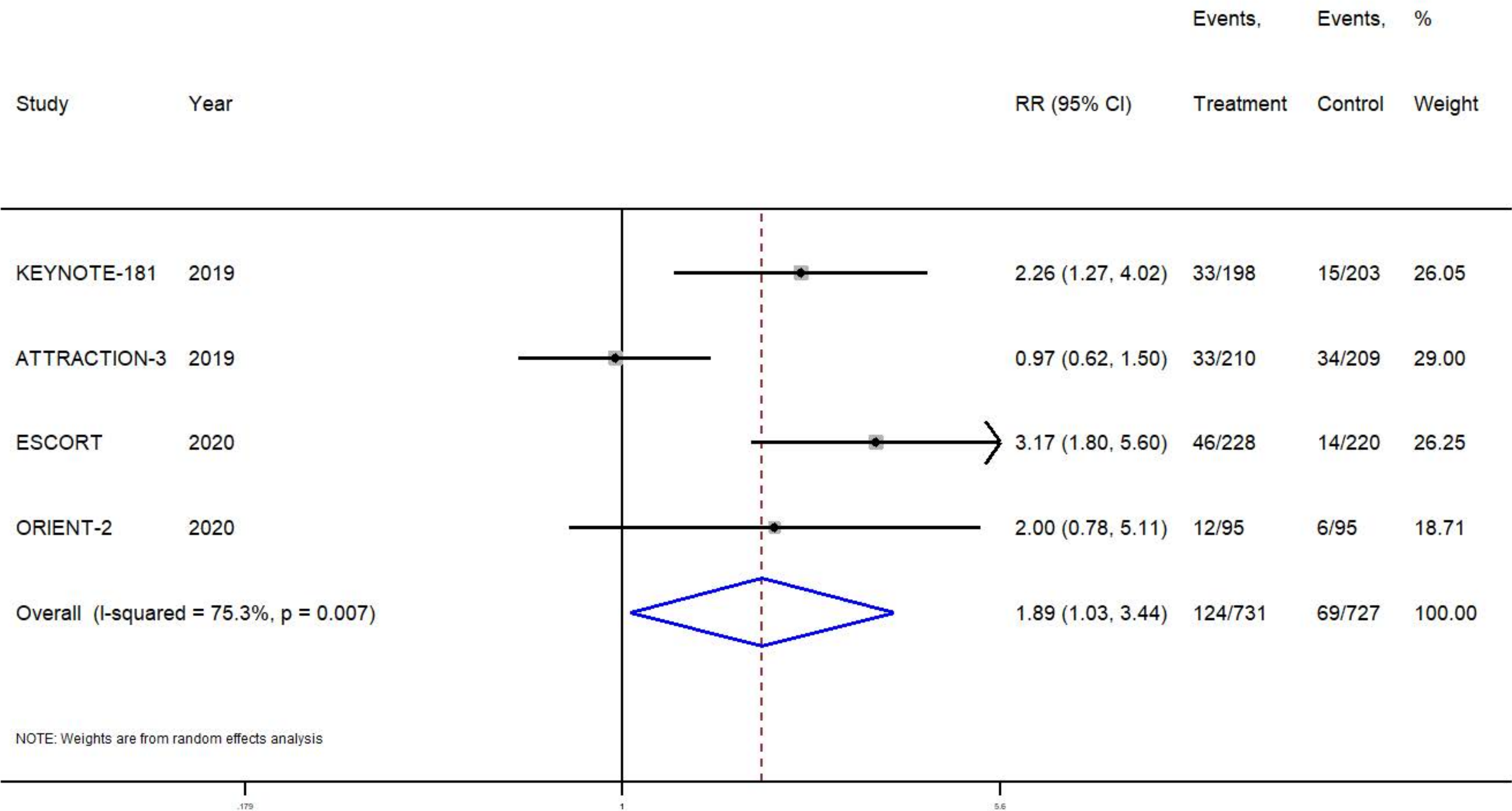

Supplement: Supplementary Materials — include Figures S1-S2 and Tables S1-S2 (see supplementary documents for details). Figure S1: Forest plot of risk ratios for objective response rate (ORR) between PD-1/PD-L1 inhibitors and chemotherapy/placebo in subgroups: (a) ORR in the squamous cell carcinoma subgroup; (b) ORR in the adenocarcinoma subgroup. Figure S2: Forest plot of hazard ratios for progression-free survival (PFS) between PD-1/PD-L1 inhibitors and chemotherapy/placebo in subgroups: (a) PFS in the squamous cell carcinoma subgroup; (b) PFS in the adenocarcinoma subgroup. Table S1: Any grade treatment-related adverse events of the PD-1/PD-L1 inhibitor group versus the chemotherapy group. Table S2: Grades 3–5 of treatment-related adverse events of the PD-1/PD-L1 inhibitor group versus the chemotherapy group. [file 3048974.f1.zip › Figure S1a.pdf]

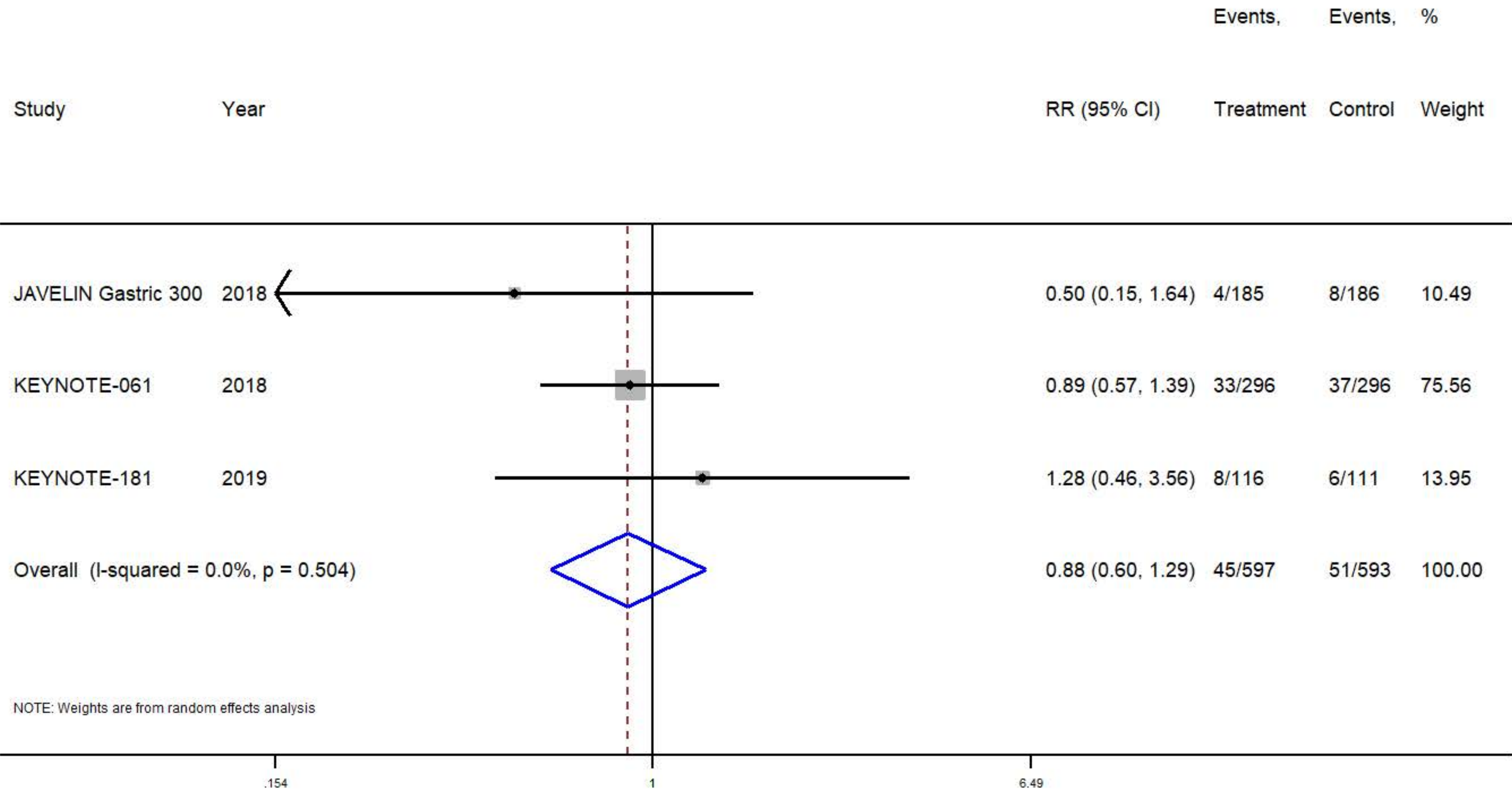

Supplement: Supplementary Materials — include Figures S1-S2 and Tables S1-S2 (see supplementary documents for details). Figure S1: Forest plot of risk ratios for objective response rate (ORR) between PD-1/PD-L1 inhibitors and chemotherapy/placebo in subgroups: (a) ORR in the squamous cell carcinoma subgroup; (b) ORR in the adenocarcinoma subgroup. Figure S2: Forest plot of hazard ratios for progression-free survival (PFS) between PD-1/PD-L1 inhibitors and chemotherapy/placebo in subgroups: (a) PFS in the squamous cell carcinoma subgroup; (b) PFS in the adenocarcinoma subgroup. Table S1: Any grade treatment-related adverse events of the PD-1/PD-L1 inhibitor group versus the chemotherapy group. Table S2: Grades 3–5 of treatment-related adverse events of the PD-1/PD-L1 inhibitor group versus the chemotherapy group. [file 3048974.f1.zip › Figure S1b.pdf]

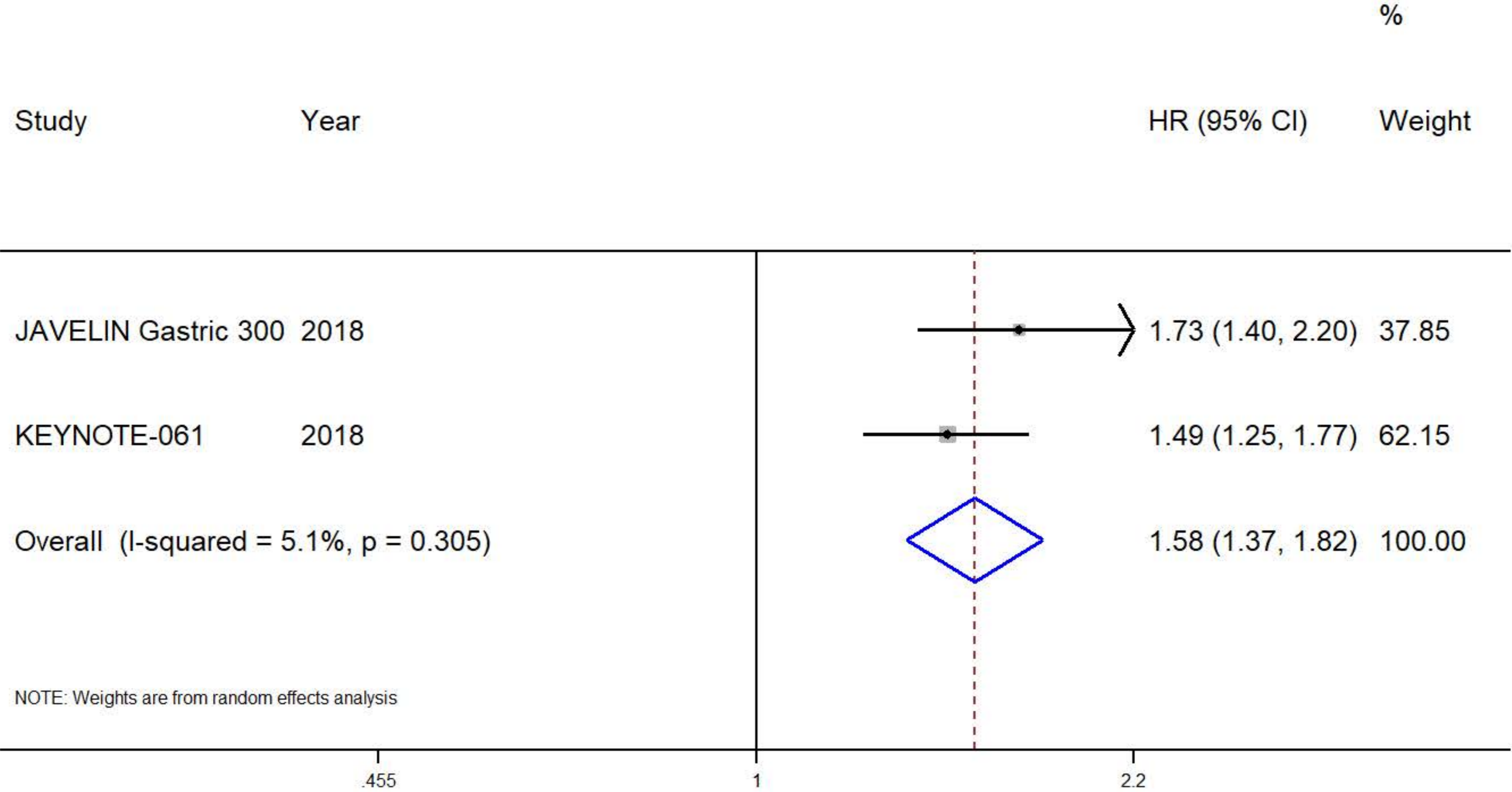

Supplement: Supplementary Materials — include Figures S1-S2 and Tables S1-S2 (see supplementary documents for details). Figure S1: Forest plot of risk ratios for objective response rate (ORR) between PD-1/PD-L1 inhibitors and chemotherapy/placebo in subgroups: (a) ORR in the squamous cell carcinoma subgroup; (b) ORR in the adenocarcinoma subgroup. Figure S2: Forest plot of hazard ratios for progression-free survival (PFS) between PD-1/PD-L1 inhibitors and chemotherapy/placebo in subgroups: (a) PFS in the squamous cell carcinoma subgroup; (b) PFS in the adenocarcinoma subgroup. Table S1: Any grade treatment-related adverse events of the PD-1/PD-L1 inhibitor group versus the chemotherapy group. Table S2: Grades 3–5 of treatment-related adverse events of the PD-1/PD-L1 inhibitor group versus the chemotherapy group. [file 3048974.f1.zip › Figure S2b.pdf]
